# Poloxamer-188 Adjuvant Efficiently Maintains Adaptive Immunity of SARS-CoV-2 RBD Subunit Vaccination through Repressing p38MAPK Signaling

**DOI:** 10.3390/vaccines10050715

**Published:** 2022-05-02

**Authors:** Chao-Hung Chen, Yu-Jen Lin, Li-Ting Cheng, Chien-Hung Lin, Guan-Ming Ke

**Affiliations:** 1 Graduate Institute of Animal Vaccine Technology, College of Veterinary Medicine, National Pingtung University of Science and Technology, Pingtung 10650, Taiwan; cch1120@mail.npust.edu.tw (C.-H.C.); m10724011@mail.npust.edu.tw (Y.-J.L.); chenglt@mail.npust.edu.tw (L.-T.C.); 2 General Research Service Center, National Pingtung University of Science and Technology, Pingtung 91201, Taiwan; 3 Country Best Biotech Co., Ltd., Taipei 100411, Taiwan; j10985001@mail.npust.edu.tw

**Keywords:** SARS-CoV-2 vaccine, poloxamer 188, p38MAPK

## Abstract

Poloxamer-188 (P188) is a nonionic triblock linear copolymer that can be used as a pharmaceutical excipient because of its amphiphilic nature. This study investigated whether P188 can act as an adjuvant to improve the immunogenicity of severe acute respiratory syndrome coronavirus 2 (SARS-CoV-2) receptor binding domain (RBD) subunit vaccine. BALB/c mice were vaccinated twice with the RBD antigen alone or in combination with P188 or MF59 (a commercial adjuvant for comparison purposes). The resulting humoral and cellular immunity were assessed. Results showed that P188 helped elicit higher neutralizing activity than MF59 after vaccination. P188 induced significant humoral immune response, along with type 1 T helper (Th1) and type 2 T helper (Th2) cellular immune response when compared with MF59 due to repressing p38MAPK phosphorylation. Furthermore, P188 did not result in adverse effects such as fibrosis of liver or kidney after vaccination. In conclusion, P188 is a novel adjuvant that may be used for safe and effective immune enhancement of the SARS-CoV-2 RBD antigen.

## 1. Introduction

COVID-19 is a novel coronavirus (SARS-CoV-2) that causes severe respiratory syndromes and was first identified in 2019 [1]. The virus spread rapidly throughout the entire world, resulting in deaths and huge economic loses [2]. Vaccination played a crucial role in ameliorating the COVID-19 pandemic [3]. SARS-CoV-2 is an enveloped RNA virus and uses the spike protein (S) on viral envelope to attach to cellular receptors such as angiotensin-converting enzyme 2 (ACE2) and Transmembrane serine protease 2 (TMPRSS2) to enter human cells (especially lung cells), leading to severe pneumonia [4]. Because of receptor specificity for ACE2 and TMPRSS2, SARS-CoV-2 can also induce severe damages to various organs [5]. The spike protein is a crucial antigen for the development of COVID-19 vaccines, including subunit vaccines, vectored vaccines, and mRNA vaccines [6,7]. The first vaccines to market were vectored SARS-CoV-2 vaccines and mRNA vaccines, which were novel designs, and only a few numbers of countries can produce them at scale. The more common technology of subunit vaccines may be a better choice for developing countries.

After the widespread use of the novel vaccines, technical problems started to surface, such as instability of the mRNA vaccines [8] and rare severe side effects of the recombinant adenovirus-vectored vaccine [9]. Since subunit vaccines can be stable, low cost, and have a standardized manufacturing process, a SARS-CoV-2 RBD subunit vaccine can serve as an alternative [10]. After vaccination with the RBD vaccine, short exposure of the RBD antigen to the body is known to insufficiently elicit effective humoral immune response, and, therefore, multiple shots or long duration adjuvants may be necessary for full immune response [11]. Eliciting and maintaining high, effective, and long-lasting titers of neutralizing antibodies is crucial to the development of commercial SARS-CoV-2 vaccines [12]. MF59 is a commercially available oil-in-water emulsion adjuvant for subunit vaccines [13]. Recently, MF59 has been shown to improve the immune response of SARS-CoV-2 subunit vaccines [14]. However, studies also reported that MF59-adjuvanted influenza vaccines may have induced autoimmune/inflammatory syndromes [15] and vasculitis [16]. In addition, recent study found that alum-adjuvanted coronavirus RBD vaccines could enhance Th2-type immune response, leading to eosinophilic pulmonary infiltration under coronavirus challenge [17].

Poloxamer-188 (P188) is a safe, nonionic triblock copolymer composed of a central hydrophobic chain of poly(propylene oxide) sandwiched between two hydrophilic chains of poly(ethylene oxide), giving it an amphiphilic nature useful as an emulsifier [18,19,20]. P188 shows excellent solubility, clarity, and concentration-dependent viscosity in aqueous solution [21]. When used as a pharmaceutical excipient, P188 exhibits lower cytotoxicity because of its ability to protect cells against peroxidation of membrane, damages of cytoskeleton, and apoptosis [22,23,24,25]. In addition, a study also demonstrated that P188 can provide neuron protection by inhibiting p38 mitogen-activated protein kinase (p38MAPK) [26]. Interestingly, p38MAPK inhibitors can be applied as an adjuvant to improve Th1 immune responses after vaccination [27].

Given the multiple useful characteristics of P188, we aimed to evaluate whether it may serve as an effective adjuvant for the SARS-CoV-2 RBD subunit vaccine. Mice were vaccinated twice with the RBD antigen alone or in combination with P188 or MF59. Antibody and cellular immune responses were examined.

## 2. Materials and Methods

### 2.1. Vaccine Formulations and Immunization of Mice

For vaccine formulation per dose, 1 or 5 μg of SARS-CoV-2 RBD (S1 + S2 fragments) was combined with 50 μL of P188 (0.04 mM, Sigma, St. Louis, MO, USA) or 50 μL of MF59 (Sigma-Aldrich, St. Louis, MO, USA). Then, 6-week-old male BALB/c mice were intramuscularly immunized twice 3 weeks apart. After vaccination, ELISA was performed to examine the levels of glutamate pyruvate transaminase (GPT), creatinine (Cr), blood urea nitrogen (BUN), neutralizing antibody titer, and plasma cytokines on Day 49. The mice were then sacrificed by CO_2_ euthanasia for pathological analysis and splenocyte isolation. 

### 2.2. Neutralization Assay Using SARS-CoV-2 Pseudoviruses

Human ACE (hACE) gene-transfected HEK293 cells (National Health Research Institutes, Miaoli, Taiwan) were seeded and incubated overnight in 96-well plates. Sera from vaccinated mice were diluted by MEM containing 1% FBS, and then mixed with an equal volume of SARS-CoV-2 pseudoviruses (National Health Research Institutes, Miaoli, Taiwan) expressing full-length wild-type Wuhan-Hu-1 strain SARS-CoV-2 spike protein. After incubation at 37 °C for 1 h, the sera-virus mixture was then added to the hACE-transfected HEK293 cells. After 1 h incubation at 37 °C, the virus-containing medium was then replaced with fresh medium and the cells were incubated for 72 h. Relative luciferase units (RLU) from lysed hACE-transfected HEK293 cells were measured by Tecan i-control (Infinite 500, Tecan Group Ltd., Männedorf, Switzerland). The valence of neutralizing antibody is presented as the reciprocal of ID_50_.

### 2.3. Binding Assay of S-Protein and Human ACE2

To establish a human ACE2 (hACE2)-overexpressing cell line, human ACE2 (NM_021804) cDNA was inserted into the Sgfl/Mlul site of the pCMV6-GFP expression vector plasmid (OriGene Technologies, Inc., Rockville, MD, USA). The resulting pCMV6-ACE2-GFP was then transfected into HepG2 cells (ATCC HB-8065) using Lipofectamine 2000 (Invitrogen, Carlsbad, CA, USA). Cells were cultured in Opti-MEM (Invitrogen, Carlsbad, CA, USA) at 37 °C for 5 h and then placed in freshly changed culture medium. The diluted sera from vaccinated mice were mixed with SARS-CoV-2 pseudovirus and incubated at 37 °C for 1 h before adding to hACE2-transfected HepG2 cells in 48-well culture plate for a 24-h incubation period in DMEM (Invitrogen, Carlsbad, CA, USA) at 37 °C, 5% CO_2_. To detect S-protein immunoprecipitated by hACE, hACE of cell lysate was first immunoprecipitated by hACE monoclonal antibody (Santa Cruz Biotechnology Inc., Santa Cruz, CA, USA) with Protein G plus/Protein A agarose beads (Santa Cruz Biotechnology Inc., Santa Cruz, CA, USA). Thereafter, the precipitates were blotted with monoclonal anti-S-protein antibody (Sigma-Aldrich, St. Louis, MO, USA). 

### 2.4. Cytokine and Apoptosis Assays 

After secondary vaccination, levels of plasma Interferon-γ (IFN-γ), Interleukin 2 (IL-2), Interleukin 4 (IL-4), and Interleukin 10 (IL-10) were determined by enzyme-linked immunosorbent assay (ELISA) purchased from MyBioSsource (San Diego, CA, USA). Isolation of primary splenocytes from spleens of vaccinated mice was conducted using the Spleen Dissociation Kit (Miltenyi Biotec, Bergisch Gladbach, Germany). Splenocytes were adherently cultured in 96-well plates containing DMEM (Invitrogen, Carlsbad, CA, USA) supplemented with 10% fetal bovine serum (Invitrogen, Carlsbad, CA, USA), 2-mercaptoethanol (Invitrogen, Carlsbad, CA, USA), 0.01% penicillin G (Sigma-Aldrich, St. Louis, MO, USA), gentamycin (Sigma-Aldrich, St. Louis, MO, USA), 0.01% HEPES (Sigma-Aldrich, St. Louis, MO, USA), and L-arginine (Sigma-Aldrich, St. Louis, MO, USA). Splenocytes were stimulated with RBD or RBD plus P188 (or MF59) for the detection of IFN-γ, IL-2, IL-4, and IL-10 mRNA and protein expression. 

Human peripheral blood mononuclear Cells (PBMCs) from Kaohsiung Medical University (Kaohsiung, Taiwan), were cultured in 96-well plates containing RPMI-1640 medium (Invitrogen, Carlsbad, CA, USA) and P188 (0, 0.02, 0.04, 0.08, or 0.1 mM) with RBD (5 μg) stimulation for 24 h. IFN-γ was measured by quantitative reverse transcription PCR (RT-qPCR) with qPCR primer: F:5′-GAGTGTGGAGACCATCAAGGAAG-3′ and R:5′-TGCTTTGCGTTGGACATTCAAG TC-3′, and apoptosis was detected by ApoStrand™ ELISA Apoptosis Detection Kit (Enzo Life Sciences, Inc., Farmingdale, NY, USA) according to manufacturer’s protocol. 

### 2.5. RT-qPCR

Total RNA from splenocytes cell lysate was extracted with Trizol (Invitrogen, Carlsbad, CA, USA) and converted to cDNA with the Super Script III cDNA Synthesis Kit (Invitrogen, Carlsbad, CA, USA). IFN-γ, IL-2, IL-4, and IL-10 cDNA were amplified using the SYBR Green I qPCR master mix kit (OriGene Technologies, Inc., Rockville, MD, USA) and qPCR primers. Respective forward and reverse primers (OriGene Technologies, Inc., Rockville, MD, USA) were as follows: qPCR primer included IFN-γ: F:5′-CAGCAACAGCA AGGCGAAAAAGG-3′ and R:5′-TTTCCGCTTCCTGAG GCTGGAT-3′; IL-2: F:5′-GCGGCATGTTCTGGATTTGACTC-3′ and R:5′-CCACCACAGT TGCTGACTCATC-3′; IL-4: F:5′-ATCATCGGCATTTTGAACGAGGTC-3′ and R:5′-ACCTTGGAAGCCCTACA GACGA-3′, IL-10: F:5′-CGGGAAGACAATAACTGCACCC-3′ and R:5′-CGGTTAGC AGTATGTTGTCCAG C-3′. Data analysis was performed by Foldchange = 2 (∆Ct treatment-∆Ct control).

### 2.6. Western Blotting

Proteins of primary splenocytes were extracted with the M-PER Mammalian Protein Extraction Reagent (Thermo Fisher Scientific Inc., Waltham, MA, USA) and were then separated with SDS-PAGE. The separated proteins on SDS-PAGE were transferred onto a PVDF membrane (Merck Millipore, Darmstadt, Germany). Then, the PVDF membrane was blocked with Tris-buffered saline with 0.2% Tween 20 (TBS-T) containing 5% skim milk at 4 °C overnight. For protein detection, the PVDF membrane was incubated with diluted primary antibodies. After washing with TBST, the PVDF membrane was incubated with a 1:10,000 dilution of horseradish peroxidase-conjugated secondary antibody in TBS-T containing 5% skim milk. Western blots were detected using the ECL Detection Kit (Merck Millipore, Darmstadt, Germany). In this experiment, anti-p-p38, p38, IFN-γ, IL-2, IL-4, and IL-10 antibodies (Santa Cruz Biotechnology, Santa Cruz, CA, USA) were used. 

### 2.7. Statistical Analysis

The GraphPad Prism software (GraphPad Software, Inc., La Jolla, CA, USA) was used for statistical analysis. Data are presented as mean ± SEM and significant difference between groups were calculated using one-way ANOVA and paired *t* test with the Bonferroni test. 

## 3. Results

### 3.1. Subsection

#### 3.1.1. P188 Enhanced the Production of Neutralizing Antibody

**Figure 1:**
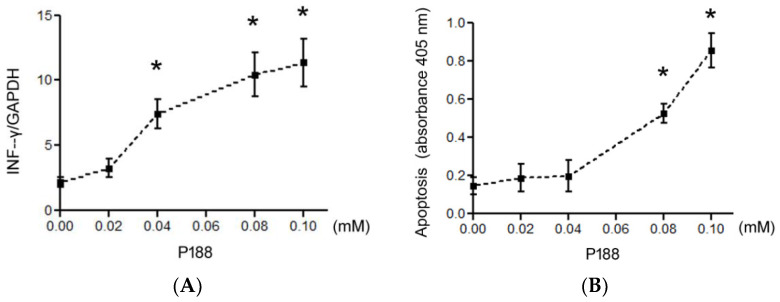
Dose-responsiveness of P188 affected expression in human PBMCs. RBD stimulated P188 (0, 0.02, 0.04, 0.08, or 0.1 mM)-cultured PBMCs for 24 h. (**A**) RT-qPCR showed P188 (0.04, 0.08, 0.1 mM) significantly increased IFN-γ. (**B**) Apoptosis assay showed P188 (0.08 and 0.1 mM) significantly triggered apoptosis. All results were presented as mean ± SD. * *p* < 0.05 vs. the control group (0 mM).

To confirm dose-responsive immune response and safety of P188, human PBMC were cultured by P188 (0, 0.02, 0.04, 0.08, or 0.1 mM) and stimulated RBD (5 μg) for 24 h. RT-qPCR showed that P188 (0.04, 0.08, and 0.1 mM) significantly enhanced TNF-γ (Figure 1A). However, P188 (0.08 and 0.1 mM) significantly induced apoptosis of PBMC (Figure 1B.)

**Figure 2:**
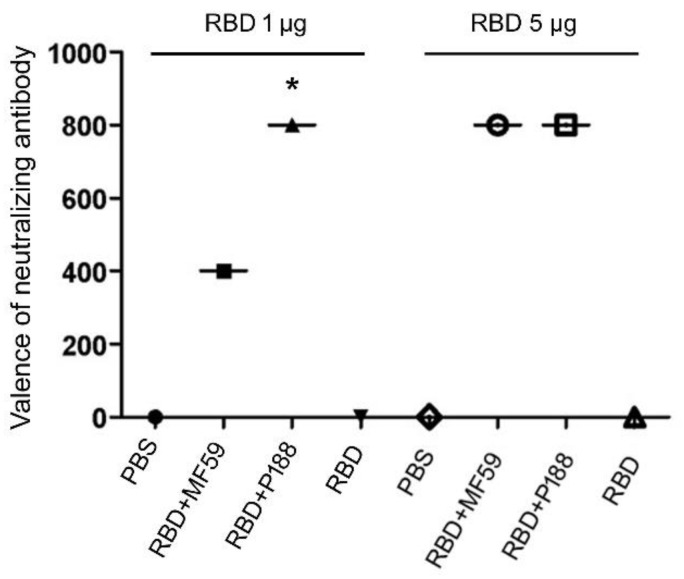
P188 significantly enhanced neutralizing antibodies after SARS-CoV-2 RBD vaccination. Neutralizing titers (reciprocal of RLU) of diluent sera from PBS; RBD-vaccinated mice (RBD); RBD and MF59-vaccinated mice (RBD + MF59); and RBD and P188-vaccinated mice (RBD + P188) was detected in vitro. The bar graph illustrates neutralizing titers of diluent sera after two-dose vaccination. All results were presented as mean ± SD. * *p* < 0.05 vs. the PBS group, and RBD + MF59 group.

To evaluate the adjuvant effect, mice were vaccinated twice with the RBD antigen (1 or 5 μg) alone or in combination with P188 or MF59. Neutralization assay using pseudoviruses showed that P188 increased the titer of neutralizing antibody in groups of 1 μg RBD immunization when compared to the MF59-adjuvanted group (Figure 2). When 5 μg of RBD was used at the antigen, there was no significant difference in neutralizing titers between the P188- and MF59-adjuvanted groups (Figure 2). In addition, liver inflammatory marker (GPT) and renal functional marker (Cr and BUN) were not up regulated for the experimental groups, indicating the safety of the adjuvants (Table 1).

#### 3.1.2. P188-Adjuvanted RBD Vaccine Induced Antibodies That Block S Protein-hACE Interaction

**Figure 3:**
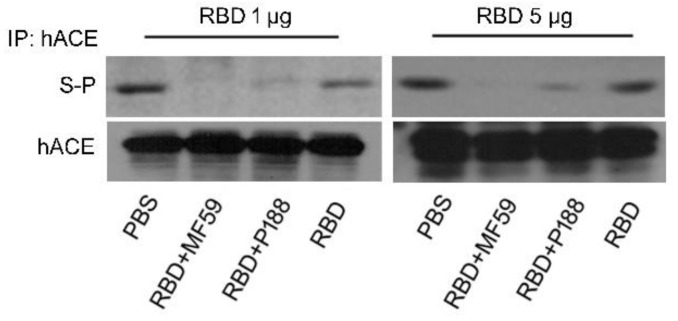
Sera of SARS-CoV-2 RBD vaccinated mice reduced S-protein binding activity on hACE overexpressing hepatic cells. Immunoprecipitation showed that sera of SARS-CoV-2 RBD vaccinated mice significantly repressed S-protein binding to hACE in vitro. Bar graphic presented that S-protein binding activity was highly repressed in group of RBD and P188-vaccination (RBD + P188) compared with group of RBD and MF59-vaccination (RBD + MF59).

To verify the binding specificity of the antibodies elicited by the vaccines, sera of vaccinated mice were evaluated for the ability to block the interaction between recombinant S protein and hACE expressed on cells. Co-immunoprecipitation analysis showed that antibodies elicited by adjuvanted RBD vaccines successfully disrupted S protein binding to hACE (Figure 3) while antibodies from the RBD-alone group do so to a less extent. Furthermore, antibodies from the P188-adjuvanted RBD vaccine group more efficiently inhibited S protein binding than those of the MF59-adjuvnated group. These results suggest that P188 helped increase the production of S protein-specific antibody.

#### 3.1.3. Splenocytes of the P188-Adjuvanted RBD Vaccine Group Produces Long-Lasting IFN-γ, IL-2, IL-10, and IL-4 

**Figure 4:**
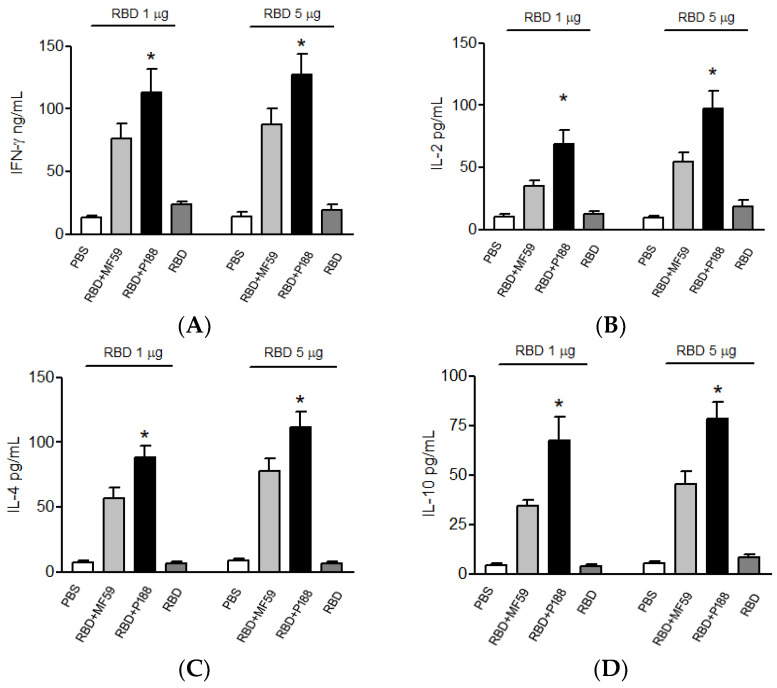
P188 up-regulated IFN-γ, IL-2, IL-10, and IL-4 in plasma of vaccinated mice compared with MF59. Plasma (**A**) IFN-γ, (**B**) IL-2, (**C**) IL-10, and (**D**) IL-4 from mice with PBS (as control), RBD plus MF59 (or P188) vaccination, and RBD alone vaccination were detected by ELISA. All results were presented as mean ± SD. * *p* < 0.05 vs. the RBD group and RBD + MF59 group.

**Figure 5:**
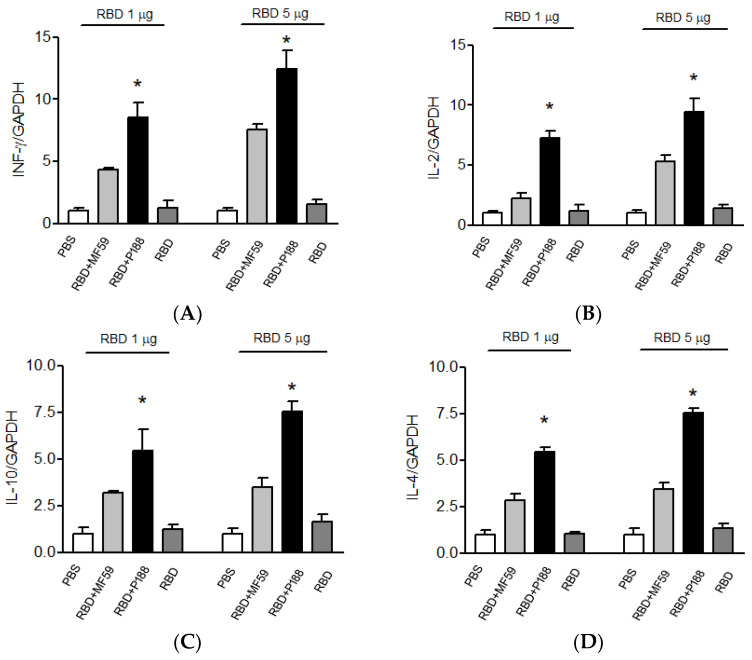
Evaluation of P188 effects on expressions of IFN-γ, IL-2, IL-10, and IL-4 mRNA in primary splenocytes of vaccinated mice. Splenocytes from vaccinated mice were cultured and stimulated by RBD for 6 h, and then evaluated. (**A**) IFN-γ and (**B**) IL-2, (**C**) IL-10 and (**D**) IL-4 mRNA expressions by RT-qPCR. Bar graphics show (**A**) IFN-γ and (**B**) IL-2, (**C**) IL-10 and (**D**) IL-4 cDNA expression in splenocytes of RBD plus P188-vaccineated mice were higher than other groups. All results are presented as mean ± SD. * *p* < 0.05 vs. the RBD groups and RBD + MF59 group.

After immunization, mice splenocytes were isolated and evaluated for cytokine expression. Compared to the MF59-adjuvanted group, splenocytes from the P188-adjuvanted group synthesized higher levels of T-helper 1-type cytokines, IFN-γ (Figure 4A) and IL-2 (Figure 4B). Furthermore, P188 also enhanced the production of T-helper 2-type cytokines, IL-10 (Figure 4C) and IL-4 (Figure 4D), when compared to MF59. These results confirmed that P188 can significantly elevate immune responses. On Day 49 after initial immunization, the memory of adaptive immunity was evaluated by measuring IFN-γ, IL-2, IL-4, and IL-10 mRNA production from splenocytes of vaccinated mice. Under RBD stimulation, splenocytes of the P188-adjuvanted group still synthesized more mRNA of T-helper 1-type cytokines, IFN-γ (Figure 5A) and IL-2 (Figure 5B) and T-helper 2-type cytokines, IL-10 (Figure 5C) or IL-4 (Figure 5D) than splenocytes of the MF59-adjuvanted group. These results confirmed that P188 provides long-lasting immune enhancement.

#### 3.1.4. P188 Enhanced IFN-γ, IL-2, IL-10, and IL-4 Expression in Splenocytes by Inhibiting p38MAPK

**Figure 6:**
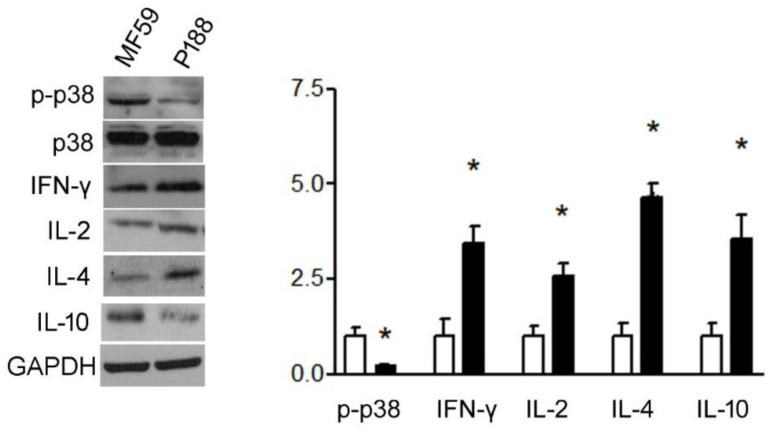
P188 up-regulated T-helper type 1 and 2 activity and down-regulated p38MAPK phosphorylation. Splenocytes from mice were cultured and stimulated by P188. Western blot and the bar graphic show that P188 repressed p-p38MAPK increase and significantly Th 1 cytokines, IFN-γ, and IL-2; whereas Th2 cytokines, IL-10, and IL-4 increased in splenocytes. All results are presented as mean ± SD. * *p* < 0.05 vs. control group.

**Figure 7:**
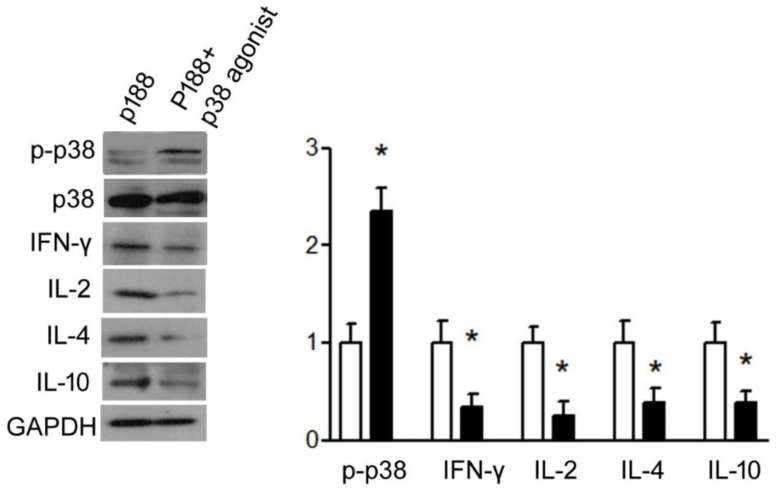
The p38 agonist diminished P188 effects on T-helper type 1 and 2 activity. Splenocytes from mice were cultured and stimulated by P188 with or without p38 agonist. Western blots and bar graph showed that Th 1 cytokines, IFN-γ and IL-2, and Th2 cytokines, IL-10 and IL-4, decreased in P188 and p38 agonist-treated group. All results are presented as mean ± SD. * *p* < 0.05 vs. P188 group.

To determine whether p38MAPK may play a role in P188-induced cytokine expression, Western blot analysis of p38 and the cytokines was performed. Results showed that P188 significantly repressed p38MAPK phosphorylation and enhanced IFN-γ and IL-2 and T-helper type 2 cytokines, IL-10, or IL-4 in primary splenocytes of mice (Figure 6). For verification, when a p38MAPK agonist, isoproterenol, is added, significant inhibition of IFN-γ, IL-2, IL-10, and IL-4 production can be seen even when P188 is present (Figure 7). 

#### 3.1.5. P188 Did Not Induce Systemic Adverse Effect after Vaccination

**Figure 8:**
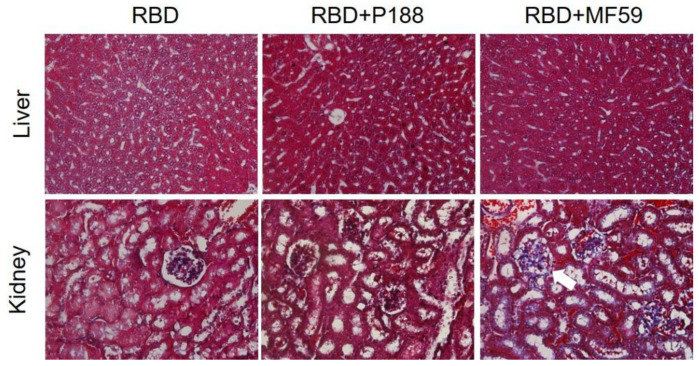
Evaluation of P188 toxicity on liver and kidney of RBD-vaccinated mice. Trichrome staining presented in liver and renal cortex sections of PBS; RBD-vaccinated mice (RBD); RBD plus MF59-vaccinated mice (RBD + MF59); and RBD plus poloxmer188-vaccinated mice (RBD + P188).

After vaccination, biochemical analysis of plasma was performed, and pathological characters of liver and kidney sections were investigated. On day 49 post vaccination, there was no mortality, abnormal body weight, and biochemical changes (ALT, BUN, creatinine) between plasma samples of PBS; RBD-vaccinated mice (RBD); MF59-vaccinated mice (RBD + MF59); and P188-vaccinated mice (RBD + P188). Figure 8 confirmed that there are no pathological lesions (Trichrome stain) in sections of liver and kidney in the P188-adjuvanted group.

## 4. Discussion

Ideally, the efficiency of controlling SARS-CoV-2 spread is reliant on vaccination with the RBD subunit antigen combined with adaptive adjuvant to stimulate innate and efficient adaptive immunities. This study showed that injection of SARS-CoV-2 RBD vaccine adjuvanted with P188 was useful to enhance neutralizing antibody against S protein-expressing the pseudovirus. P188 induced Th1 and Th2 immune responses and higher levels of neutralizing antibody than MF59 without adverse effects during in vivo studies. 

To improve the immune efficacy of SARS-CoV-2 RBD vaccines, P188 first was used for adjuvant to effectively enhanced production of anti-SARS-CoV-2 antibody for a more sustained period. In terms of the immunization schedule, we saw that immunization significantly induced anti-SARS-CoV-2 RBD antibody titer after two-dose vaccination and successfully neutralized recombinant S protein of SARS-CoV-2 in vitro. Specifically, P188 induced higher antibody titers than MF59 under the same concentration of RBD antigen vaccination in vivo study. After vaccination, we still detect higher titers of neutralizing antibody in plasma of vaccinated mice with P188-adjuvanted RBD than MF59-adjuvanted RBD during the third week. In addition, vaccination with poloxmer188-adjuvanted RBD did not significantly alter GPT, BUN, and Cr in serum, and fibrosis of liver and kidney. These studies suggest that P188 is a safe, efficient, and novel adjuvant for the SARS-CoV-2 RBD vaccine. 

For considering clinical immune responsive pathway, the adjuvant of SARS-CoV-2 RBD vaccine must be suitable for inducing sufficient humoral and cellular immune response against SARS-CoV-2 infection. A current study showed that P188 enhanced higher Th1 cytokines (IFN-γ and IL-2) and Th2 cytokines (IL-10 and IL-4) in plasma compared with MF59 after vaccination. These results pointed out that P188-induced immunization is dominant Th1 and Th2 immune response to be adjuvant of SARS-CoV-2 RBD vaccine.

P188 exhibits extremely low toxicity (oral LD_50_ 5700 mg/Kg). In clinical trials of healthy people, P188 of intravenous injection (10, 30, and 45 mg/kg/h for 72 h, or 60 and 90 mg/kg/h for 24 h) causes mild and frequent events, including back pain, leg pain, headache, and nausea. Clinical trial of patients with sickle cell disease showed P188 of intravenous therapy (30 mg/kg/h) for 72 h could induce mild and frequent events such as injection site pain, nausea, headache, vomiting, abdominal pain, or constipation [28]. In a clinical trial of children and adults with sickle cell disease, P188 of intravenous therapy (100 mg/kg) for one hour triggered adverse events including abdominal distension, hepatobiliary disorders, hyperbilirubinemia; the above adverse events were resolved in 30 days [29]. In addition, a recent pharmacological study reported that treatment of P188 alone induced the expression of IL-6 [30], suggesting P188 is potential immune stimulator. Above studies support the mechanism, application, and safety dose of P188 as potential adjuvant in formulation of subunit vaccine. In this study, intramuscular injection of SARS-CoV-2 RBD plus P188 (0.04 mM) efficiently stimulated humoral immune response without affecting hepatic inflammatory marker, GPT, and index of renal function, Cr and BUN. Sections of liver and kidney of RBD plus P188-immunized mice both showed no fibrosis. Furthermore, in vitro study demonstrated RBD plus P188 (0.04 mM) with increased tendency of IFN-γ and without significant apoptosis in human PBMCs. Therefore, 0.04 mM P188 is a safe and suitable dosage as adjuvant for SARS-CoV-2 RBD vaccine. 

Recent study confirms that P188 can keep good chemical stability in solution under environment stress such as temperature and pH alteration [25]. Moreover, P188 is also a novel reagent of biomaterials without impairing biofunction and activity [22,23,24,25]. Several studies have showed that P188 can help resist peroxidation of membrane and prevent damages of cytoskeleton and apoptosis [22,23,24]. However, very few studies have investigated the effect of P188 on immune response. This is the first study to confirm the immunological effects of P188 on Th1 and Th2 response. P188 significantly enhanced antibody titer after SARS-CoV-2 RBD vaccination compared with licensed adjuvant, MF59. In addition, P188 adjuvant significantly improved the neutralizing titers in comparison with MF59 in 1 μg RBD vaccination. In vitro, P188 could repress p38 phosphorylation and up-regulate Th1 and Th2 immune response in splenocytes through significant activation of p38MAPK compared with MF59. However, p38MAPK agonist could repress P188 effects on splenocytes of mice, suggesting that P188 is dependent on inhibiting p38MAPK activity to perfect adaptive immunity in RBD vaccinated mice. P188 is safely and wildly used in the pharmaceutical industry as an excipient of formulations and an agent of drug delivery. Based on stable packaged drug, protein, or nucleotide delivery, affinity with cell membrane, and inducible cytoprotecting of P188, a recent study focuses on medicine industrial application including being sublingual permeation enhancers [31], stabilizer of anti-tumor drug [32], agent of promising blood cell viability [33], and transfection reagent of short nucleotide chains [34]. Furthermore, our study first suggests P188 not only has the potential as an adjuvant of subunit vaccines, but also as immune modulator and drug through affecting the p38MPAK pathway.

## 5. Conclusions

Our study showed that P188 can induce better Th1 and Th2 immune responses than MF59. Co-administration of P188 with SARS-CoV-2 RBD allowed a significant boost in neutralizing antibody titer without adverse effect. This use of P188 as adjuvant is an attractive strategy for the development of commercial vaccines against COVID-19.

## Figures and Tables

**Table 1:** GPT, CR, and BUN profiles of experimental groups. Biochemical examination showed GPT, CR, and BUN did not significantly change between PBS, RBD (1 or 5 μg) plus MF59, RBD (1 or 5 μg) plus P188, and RBD-alone group. All results are presented as mean ± SD.

	1 μg RBD				5 μg RBD			
Group	PBS	RBD + MF59	RBD + P188	RBD	PBS	RBD + MF59	RBD + P188	RBD
GPT (U/I)	51.14 ± 13.12	76.11 ± 21.13	61.15 ± 19.17	59.71 ± 22.63	57.17 ± 19.11	78.37 ± 18.93	69.17 ± 14.73	75.43 ± 19.77
Blood urea nitrogen (BUN) (mg/dL)	18.24 ± 3.51	16.82 ± 6.73	18.33 ± 5.61	21.11 ± 7.37	19.43 ± 8.11	22.16 ± 7.23	19.57 ± 8.36	15.78 ± 9.05
Creatinine (Cr) (μmol/L)	17.83 ± 4.27	18.79 ± 6.53	16.93 ± 6.53	20.17 ± 4.81	23.11 ± 7.19	15.85 ± 5.67	17.74 ± 4.71	19.45 ± 4.83

## Data Availability

Not applicable.
